# Reducing image artifacts in sparse projection CT using conditional generative adversarial networks

**DOI:** 10.1038/s41598-024-54649-x

**Published:** 2024-02-16

**Authors:** Keisuke Usui, Sae Kamiyama, Akihiro Arita, Koichi Ogawa, Hajime Sakamoto, Yasuaki Sakano, Shinsuke Kyogoku, Hiroyuki Daida

**Affiliations:** 1 Department of Radiological Technology, Faculty of Health Science, Juntendo University, 2-1-1, Hongo, Bunkyo-ku, Tokyo, 113-8421 Japan; 2 Department of Radiation Oncology, Faculty of Medicine, Juntendo University, 2-1-1, Hongo, Bunkyo-ku, Tokyo, 113-8421 Japan; 3 Faculty of Science and Engineering, Hosei University, 3-7-3, Kajino, Koganei, Tokyo 184-8584 Japan

**Keywords:** Sparse projection, CGAN, Deep learning, Artifact reduction, CT, Medical imaging, Three-dimensional imaging, Tomography

## Abstract

Reducing the amount of projection data in computed tomography (CT), specifically sparse-view CT, can reduce exposure dose; however, image artifacts can occur. We quantitatively evaluated the effects of conditional generative adversarial networks (CGAN) on image quality restoration for sparse-view CT using simulated sparse projection images and compared them with autoencoder (AE) and U-Net models. The AE, U-Net, and CGAN models were trained using pairs of artifacts and original images; 90% of patient cases were used for training and the remaining for evaluation. Restoration of CT values was evaluated using mean error (ME) and mean absolute error (MAE). The image quality was evaluated using structural image similarity (SSIM) and peak signal-to-noise ratio (PSNR). Image quality improved in all sparse projection data; however, slight deformation in tumor and spine regions was observed, with a dispersed projection of over 5°. Some hallucination regions were observed in the CGAN results. Image resolution decreased, and blurring occurred in AE and U-Net; therefore, large deviations in ME and MAE were observed in lung and air regions, and the SSIM and PSNR results were degraded. The CGAN model achieved accurate CT value restoration and improved SSIM and PSNR compared to AE and U-Net models.

## Introduction

Computed tomography (CT) has been widely used for the clinical diagnosis of many organ diseases, and its clinical benefits are indispensable in modern medical strategies. However, excessive X-ray doses increase the stochastic risks of cancer at diagnostic dose levels, and the overuse of CT scans in medical environments raises concerns^1,2^. Methods for achieving the lowest radiation dose are imperative for many CT tasks^3^. This problem can be easily addressed by reducing the number of X-ray photons; however, this leads to an increase in image noise and may cause further risks of missed diagnoses. Iterative reconstruction methods combine the statistical properties of the data in the image domain and projection space to optimize the objective function and remove noise and artifacts from low-dose CT images^3,4^. This technique depends on the CT manufacturer’s specifications, which limits the clinical applications of conventional image-based denoising methods, such as total variation minimization^5^. Another effective strategy is to decrease the number of projections for a given trajectory, called the sparse-view CT technique^6–8^. Sparse projections cannot be implemented in conventional multidetector CTs because of the continuous helical acquisition of projection data. By contrast, sparse-view CT can reduce the irradiated dose in cone-beam CT (CBCT) equipped with dental CT, mammography tomosynthesis, and linear accelerator, and clinical benefits will be sufficient^9,10^. Additionally, by reducing the number of projections in 4-dimensional (4D) CBCT, a shorter imaging time can be achieved. This potential reduction in imaging time can be realized by decreasing the amount of data acquisition and processing required, thereby improving the efficiency and practicality of 4D CBCT imaging. Moreover, to generate motion artifact minimization with non-static objects during projection data acquisition, such as in dynamic CT imaging situations, sparse-view CT can be an algorithmic strategy to address them^8^. However, insufficient projection views lead to inaccurate image reconstruction and can result in prominent streak artifacts^11–13^. Therefore, various artifact reduction methods have been used to restore image quality with sparse projection data. Compressed sensing-based regularization techniques perform well on severely undersampled data^14–16^. However, these methods require prior knowledge of the image to be restored, and they successfully remove local noise but do not remove linear artifacts that appear as large structures on limited-angle tomography^17^.

Recently, with the development of deep learning in medical imaging, several approaches have been proposed to remove the noise associated with low-dose CT^18–20^. Various convolutional neural network (CNN)-based methods have been proposed; however, they often suffer from disappearing edges and image blurring^21,22^. Moreover, the streaking artifacts caused by projection reduction are difficult to remove using conventional CNNs^23^. Because these streak-shaped artifacts do not occur locally, they stretch over the image. Image-based CNNs work using hierarchical sliding convolutional windows but ultimately have a limited perceptive field of contextual information in the image. Therefore, simple CNNs are handled by sliding convolutions but are harder due to non-local effects like streaking, which lead to vanishing gradients and make training ineffective. Therefore, a skip connection with residual learning networks has been used to reduce these artifacts. A generative adversarial network (GAN) achieves accurate consistency when the underlying structures are similar even when mapped to nonlinear domains^24,25^. In particular, the conditional generative adversarial network (CGAN) algorithm has shown a superior ability to synthesize images from uniform label maps and is used as a solution to image-to-image translation^26^. Therefore, CGAN may be advantageous for reducing image artifacts in sparse-view CT using a large paired supervised image with artifacts.

In this study, we investigated the effect of image artifact correction in sparse-view CT using the CGAN model on lung CT images, based on numerous computational artifact simulation images. The accuracy of streak artifact correction was evaluated by comparing and verifying its dependence on the number of sparse projection angles. The effect of image quality correction by the CGAN-based model was compared to that of the autoencoder (AE) and U-Net models, which are widely applied for medical image correction, to clarify the image quality and improvement effect. The contribution of this study lies in demonstrating the feasibility of utilizing sparse projection CT for practical applications.

## Methods

### Image data acquisition

Lung CT images were used to train the deep learning model, and image artifacts in sparse-view CT images were simulated by forward projecting the images and subsampling the simulated projections. Lung CT images of 40 patients were obtained from a publicly available dataset, The Cancer Imaging Archive, which is an open-access information source created by the US National Cancer Institute^27^. The total number of images for these 40 patients was 4250 images. In this dataset, CT images were acquired using multidetector CT with helical scanning. The tube voltage was 120 kV, thickness of each CT image was 3 mm, matrix size was 512 × 512 pixels, and field of view 45 × 45 cm.

### Generation of simulated sparse projection image

**Figure 1:**
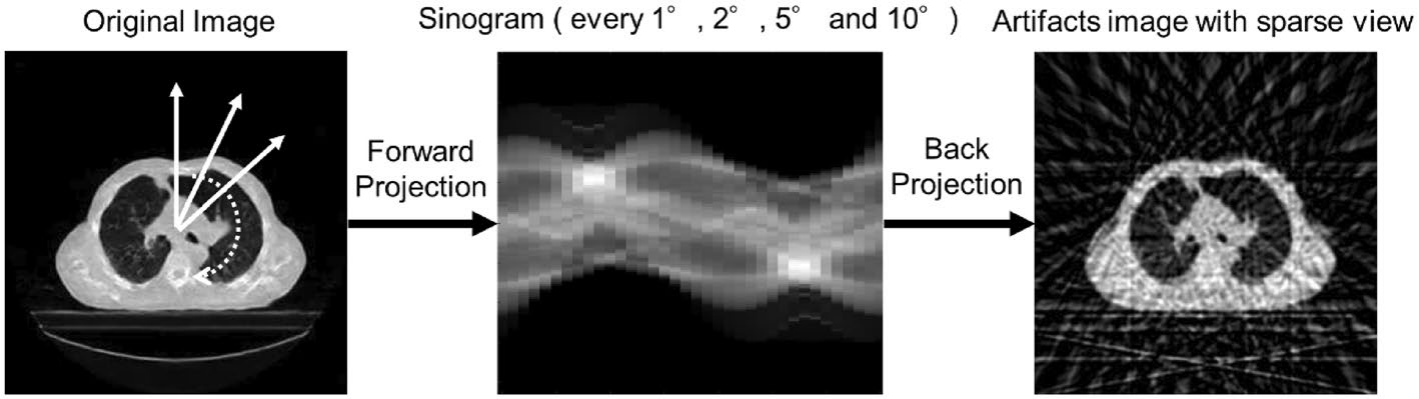
Overview of the generation of simulated sparse projection image. The sinograms of sparse projection were obtained by forward projecting to original images every 1°, 2°, 5°, and 10°. The simulated artifact image as sparse-view CT was created using the filtered back projection with the Shepp-Logan filter.

A total of 4250 simulated artifact images of the sparse projection were generated by each decimating the projection data from the original image. We obtained fan-beam projections by accumulating the CT values in each projection path at a distance of 400 pixels from the apex of the fan beam to the center of the image rotation. The spacing between arc-shaped detectors was set to 0.25°. These sparse projection data were acquired at rotational interval angles of 1°, 2°, 5°, and 10°, and a sparse sinogram was obtained using these projection data in each of the 4250 slices. Then, simulated artifact images were created from these sparse sinograms using a filtered backprojection algorithm with a Shepp and Logan filter. An overview of the generation of the simulated sparse projection image is shown in Fig. 1. In this study, we used a supervised image paired with the original and simulated images to train and evaluate the deep learning model.

### Artifacts correction in sparse-view CT by deep learning

For each deep learning model, the training dataset comprised 4250 lung CT images, with varying levels of artifacts at each dispersed projection angle. Therefore, the number of simulated artifact images was 4250 for each dispersed projection angle, i.e., 1°, 2°, 5°, and 10°. In each dispersed projection, 90% of the patient cases were used for training, and the remaining 10% were used for image quality evaluation, which were randomly selected patient cases. Therefore, the same patient data were not used for model training and evaluation. In the input data, each pixel value was normalized to (0, 1) between the maximum and minimum intensities of the pixel value per training image. An artifact-corrected image was generated using the CGAN model. Moreover, the AE and U-Net models were used to generate artifact-corrected images for comparison. The details of these deep learning models are presented below.

### CGAN

**Figure 2:**
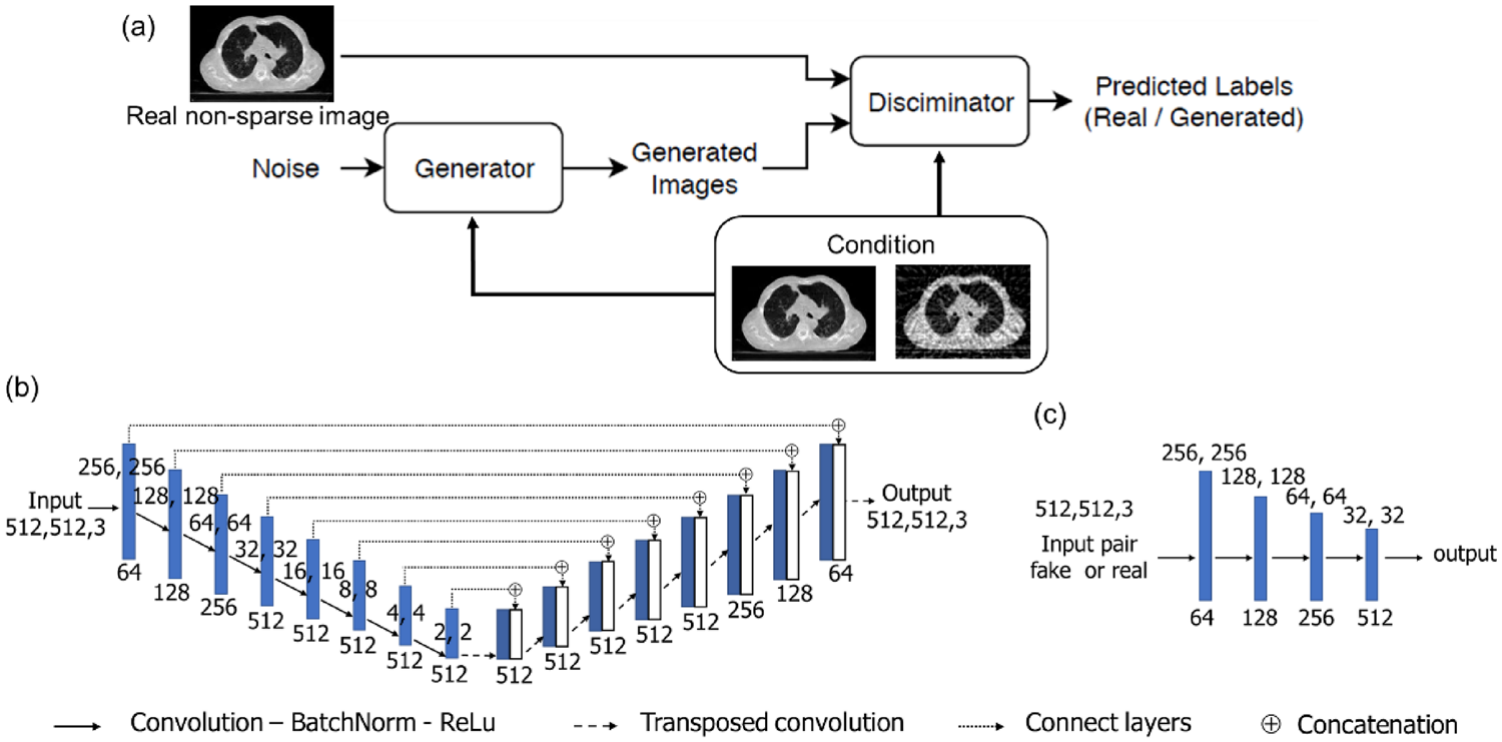
(**a**) Flowchart of conditional generative adversarial network (CGAN), and the architecture of the generator (**b**) and discriminator (**c**). The network consists of one generator and one discriminator with a conditional argument. The overall network’s performance is enhanced through each network acting bidirectionally. The artifacts in sparse projection are corrected by a network that maps images from a source domain (with artifact image) to the target domain (artifacts correction image) based on the conditional ideal image pair.

CGAN has two CNNs, one generator, and one discriminator, which have opposite functions. During the training process, the generator attempts to produce an artifact-reduced image, whereas the discriminator networks attempt to distinguish the artificially created data as either the original CT image or artificially generated. In this study, the conditional label was an image pair of the original and simulated artifact images. Figure 2 presents a flowchart of the CGAN model and the architecture of the generator and discriminator. The real non-sparse images were used as labels in the discriminator, and paired images with sparse and non-sparse images were added as conditions in the generator and discriminator. The modules of these models were in the form of convolution-BatchNorm-ReLu. Patch-GAN was utilized in the discriminator, which classified an image as either true image or generated image in each given patch. In addition, four convolutional layers were added to the discriminator architecture. The total loss function in CGAN training is as follows^26^:

1 $${L}_{cGAN}\left(G, D\right)={\mathbb{E}}_{x, y}\left[{\text{log}}D\left(x, y\right)\right]+{\mathbb{E}}_{x, z}\left[{\text{log}}\left(1-D\left(x, G\left(x, z\right)\right)\right)\right]$$

where the generator ($$G)$$ attempts to minimize the loss function ($${L}_{cGAN}\left(G, D\right)$$), whereas the discriminator ($$D$$) attempts to maximize it to distinguish between $$G\left(x, z\right)$$ and real samples $$x$$. Furthermore, estimation error loss to the discriminator feedback is added for effective training of the generator. Therefore, the final objectives were as follows:

2 $${G}^{*}=arg\,\underset{G}{{\text{min}}}\,\underset{D}{{\text{max}}}\,{L}_{cGAN}\left(G, D\right)+\lambda {L}_{{L}_{1}}\left(G\right)$$

where ${L}_{{L}_{1}}\left(G\right)$ is an additional L1-norm-based loss function in the generator to get closer to the ground-truth output $y$, and $\lambda$ is a tunable parameter; in this study, we set to 100.

We conducted experiments on a personal computer equipped with two GPUs (Quadro RTX 5000, NVIDIA Corporation) and a CPU (Intel Xeon Silver 4210R) with 96 GB of memory. The proposed algorithm was implemented using MATLAB 2022b (MathWorks Inc., Natick, MA, USA). The network was optimized using the Adam optimizer with a learning rate of 0.0002 for both the generator and discriminator networks. We used a batch size of one, and the training was stopped at 200 epochs. The loss curves of ${L}_{{L}_{1}}\left(G\right)$ approached zero as the number of epochs increased and then remained almost unchanged as they approached 200 epochs. The loss function of the generator and discriminator, as depicted in Eq. (1), were adjusted to fluctuate around 0.5 by adjusting the hyperparameters. Then, object ${G}^{*}$ shown in Eq. (2) was updated.

### AE

**Figure 3:**
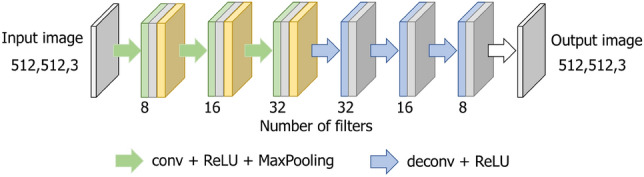
Encoder–decoder deep learning architecture of the autoencoder (AE) model. The encoder process is denoted by light green arrows and the decoder process by light blue arrows.

The AE model consisted of an encoder–decoder process with four layers^21^. Each layer has the modules of convolution-ReLu-maxPooling and reconstruction and generates an output of the same size as the input image. The filter size was 3 × 3, and the initial number of filters was eight. Figure 3 shows the network structure of AE. In this model, the input image is a simulated artifact image with sparse projection, and the output is the original image without artifacts. This network learns to match the input and output and extracts only the important information necessary for restoration from the training data. The network was optimized using the Adam optimizer with a learning rate of 0.001 and a batch size of 4. The training stopped after 200 epochs. The loss curves decreased continuously and remained almost unchanged after 200 epochs.

### U-Net

**Figure 4:**
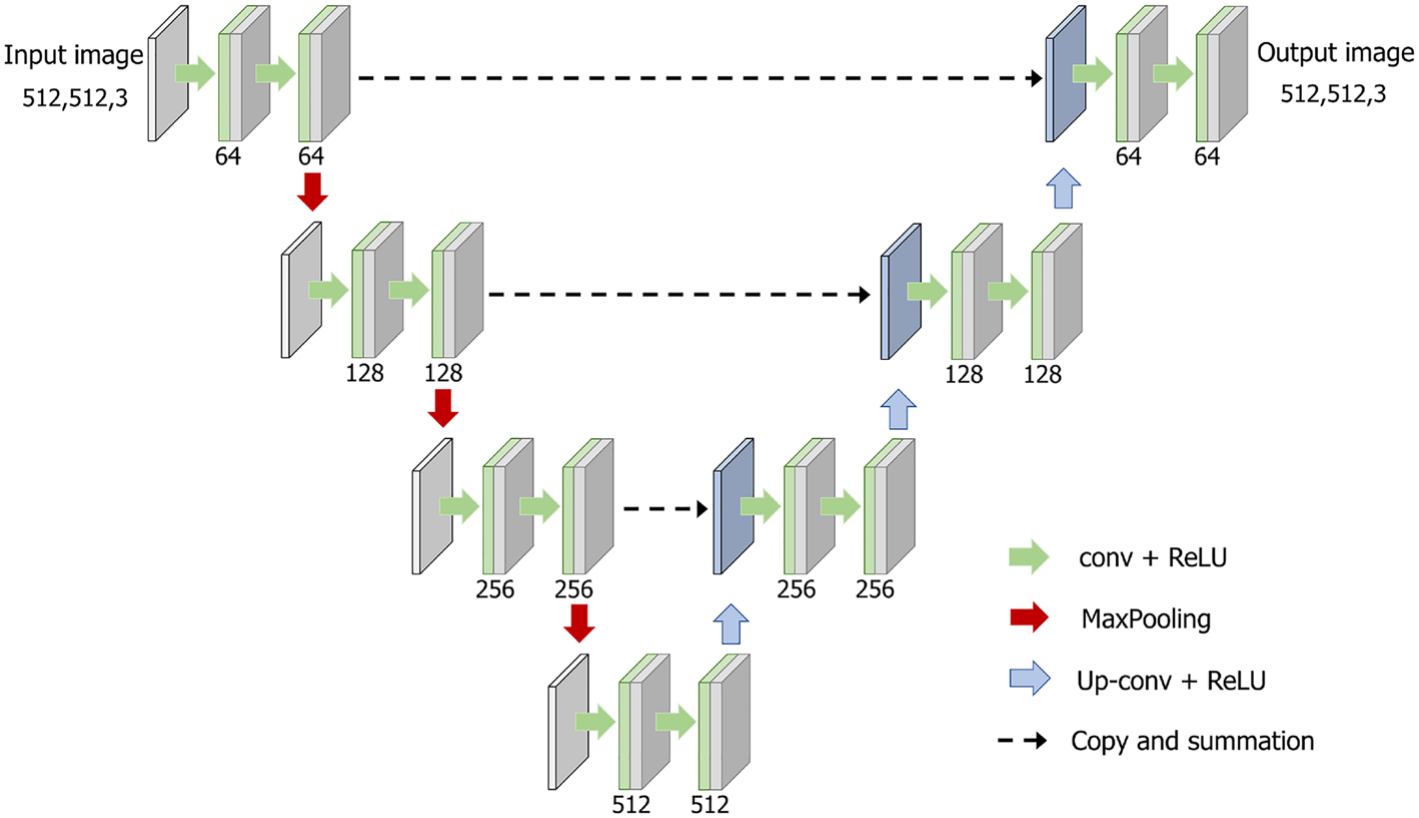
Detailed U-Net architecture used in this study. Concatenated images are shown in light blue. The channel numbers are displayed at the bottom of the image.

The U-Net model has four encoder and decoder depths, and each layer has convolution-ReLu-maxPooling modules. Skip connections are utilized in the channels at each layer to restore the overall location information while preserving local features^28^. The filter size was 3 × 3, and the number of initial filters was 64. This network comprises a context aggregation pathway that represents the input and a localization pathway that recombines these representations with shallower features. Figure 4 shows the network structure of U-Net. The input image was a simulated sparse artifact image, and the output image was the original image paired with the input. This network was optimized using the Adam optimizer with a learning rate of 0.001 and a batch size of 4. Training was stopped at 200 epochs, and the loss curves increased as the number of epochs increased and then remained almost unchanged as they approached 200 epochs.

### Image quality evaluation

#### ME and MAE

**Figure 5:**
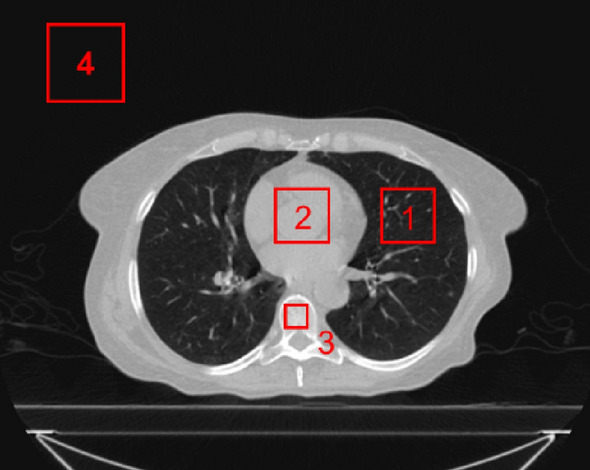
Position (1 to 4) of each ROI for calculating the mean error and mean absolute error compared with the original CT image. ROI (1 to 4) positions of the lung, soft tissue, bone, and air regions, respectively.

Test images, not used in training were selected using the original full view image as ground truth and the simulated sparse projections as model inputs. The model outputs were compared against these ground truth inputs using ME and MAE to assess streak reduction performance. The quality of the corrected images was quantitatively evaluated by comparing them with the original CT images. To evaluate the differences in the CT number with respect to the original images, we set regions of interest (ROIs) in the lung, soft tissue, bone, and air regions and measured the mean error (ME) and mean absolute error (MAE) as follows:

3 $$ME\left(X,Y\right)=\frac{1}{M\times N}\sum _{i=1}^{M}\sum_{j=1}^{N}(X(i,j)-Y(i, j))$$

4 $$MAE\left(X,Y\right)=\frac{1}{M\times N}\sum _{i=1}^{M}\sum_{j=1}^{N}\left|X(i, j)-Y(i, j)\right|$$

where $M$ and $N$ are the width and height of the pixels within an ROI, respectively, $X(i, j)$ is the CT number of the i-th and j-th pixels in the spared projection image or artifact reduction image, and $Y(i, j)$ is the CT number of the i-th and j-th pixels in the original CT image. The sizes of the ROIs were 40 × 40, 40 × 40, 15 × 15, and 50 × 50 pixels in the lung, soft tissue, bone, and air regions, respectively. An example of these ROI positions is shown in Fig. 5

#### SSIM and PSNR

To evaluate the differences in overall image quality, the structural similarity index (SSIM) and peak signal-to-noise ratio (PSNR) of the artifact-reduced image were calculated based on the original CT image^22,29^. The SSIM of images $X$ and $Y$ is defined as follows:

5 $$SSIM\left(X, Y\right) =\frac{\left(2{\mu }_{X}{\mu }_{Y}+{C}_{1}\right)\left(2{\sigma }_{XY}+{C}_{2}\right)}{\left({{\mu }^{2}}_{X}+{{\mu }^{2}}_{Y}+{C}_{1}\right)\left({{\sigma }^{2}}_{X}+{{\sigma }^{2}}_{Y}+{C}_{2}\right)}$$

where ${\mu }_{X}$ and ${\mu }_{Y}$ are the average pixel values of the image pair ($X, Y$), ${\sigma }_{X}$ and ${\sigma }_{Y}$ are the variances, ${\sigma }_{XY}$ is the covariance of $X$ and $Y$, and the $C$ terms are regularization constants, where ${C}_{1}$ equals ${\left(0.01 \times 2000\right)}^{2}$, ${C}_{2}$ equals ${\left(0.03 \times 2000\right)}^{2}$, and 2000 is the dynamic range of the images. PSNR is defined as follows:

$$PSNR=10{{\text{log}}}_{10}\frac{max{(X\left(i,j\right))}^{2}}{MSE}$$

6 $$MSE=\frac{1}{M\times N}\sum_{i=1}^{M}\sum_{j=1}^{N}{\left(X\left(i,j\right)-Y(i,j)\right)}^{2},$$

PSNR is defined as the maximum value in an input image $X\left(i,j\right)$ divided by the mean squared error between images $X$( with artifacts or the corrected image) and $Y$ (the original CT image). In addition, $M$ and $N$ represent the width and height of the images, respectively. Differences in SSIM and PSNR were evaluated as statistically significant using a two-tailed *t*-test.

### Ethical approval

All procedures performed in this study were in accordance with the ethical standards of the institution or the practice at which the study was conducted.

### Consent to participate

CT data were obtained from The Cancer Imaging Archive (http://www.cancerimagingarchive.net/).

## Results

**Figure 6:**
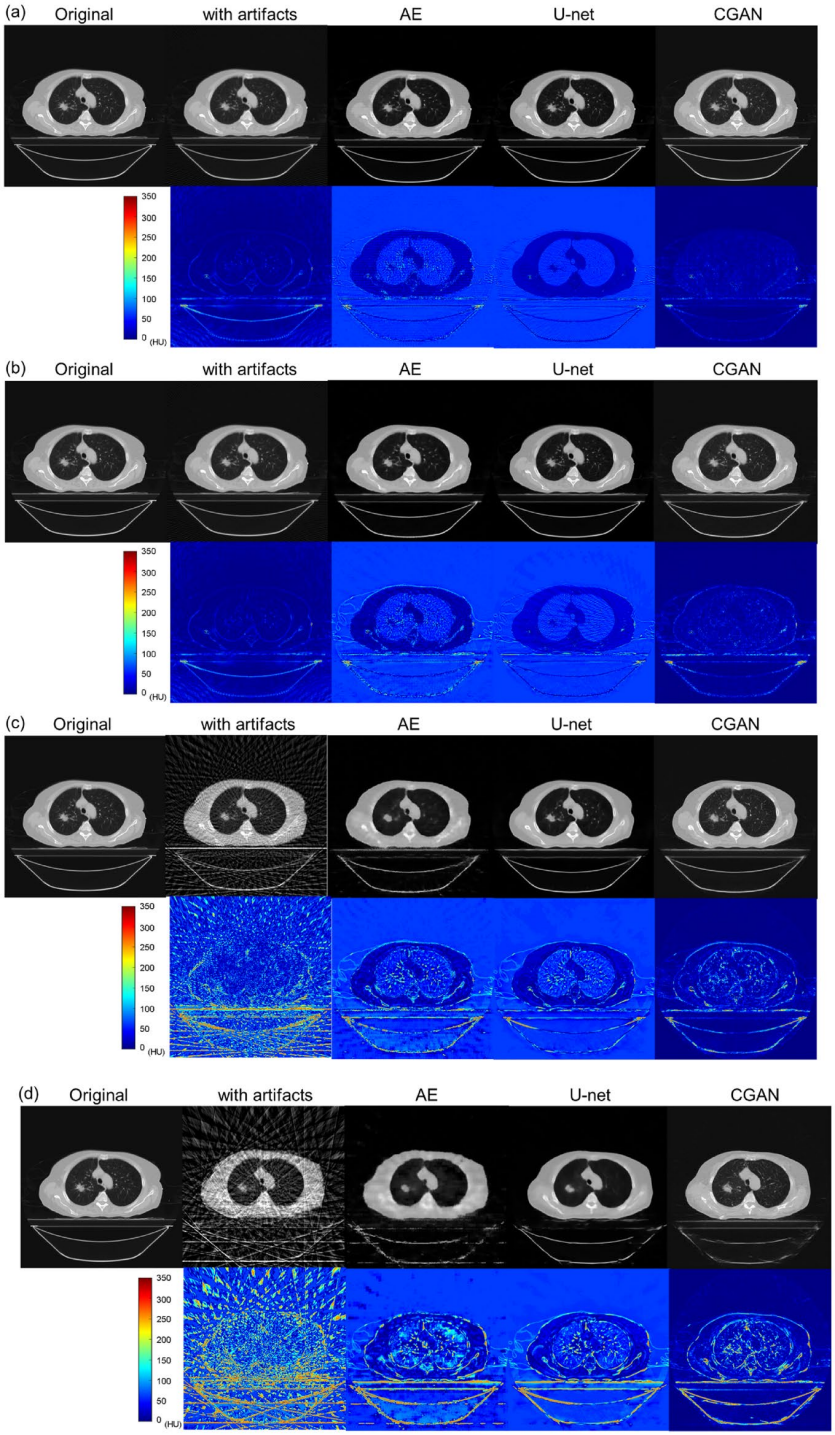
Results of artifact-corrected image in sparse-view CT by AE, U-Net, and CGAN. (**a**–**d**) Representative axial slice images with dispersed projection angles of 1°, 2°, 5°, and 10°. Subtraction image, created by subtracting the artifact-corrected image from the original image, is shown at the bottom of each dispersed projection image. All images are shown with the same window width and levels.

**Figure 7:**
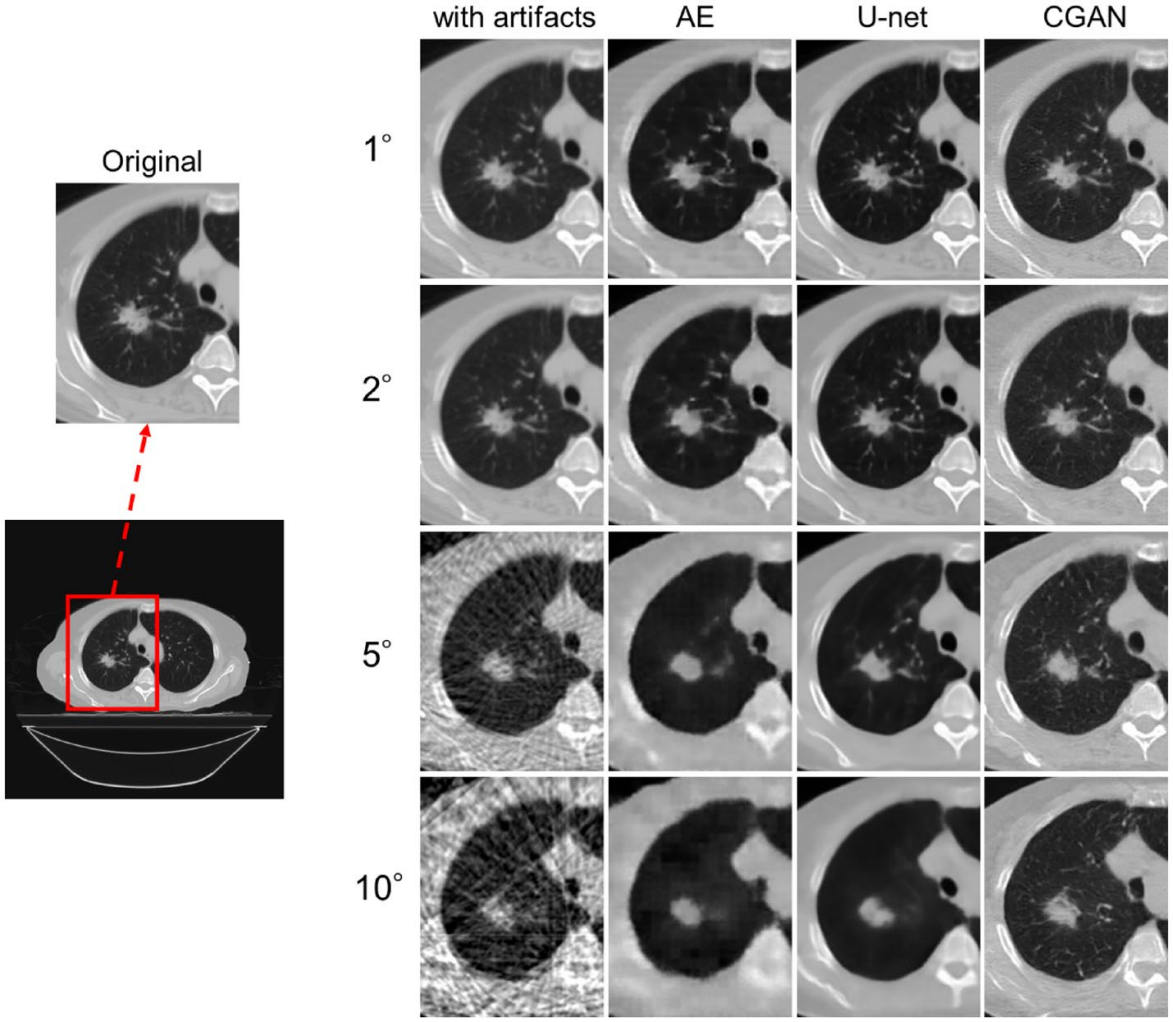
Enlarged images of the region indicated by the red region of interest with each result of sparse-view CT. All images are shown with the same window width and levels.

**Figure 8:**
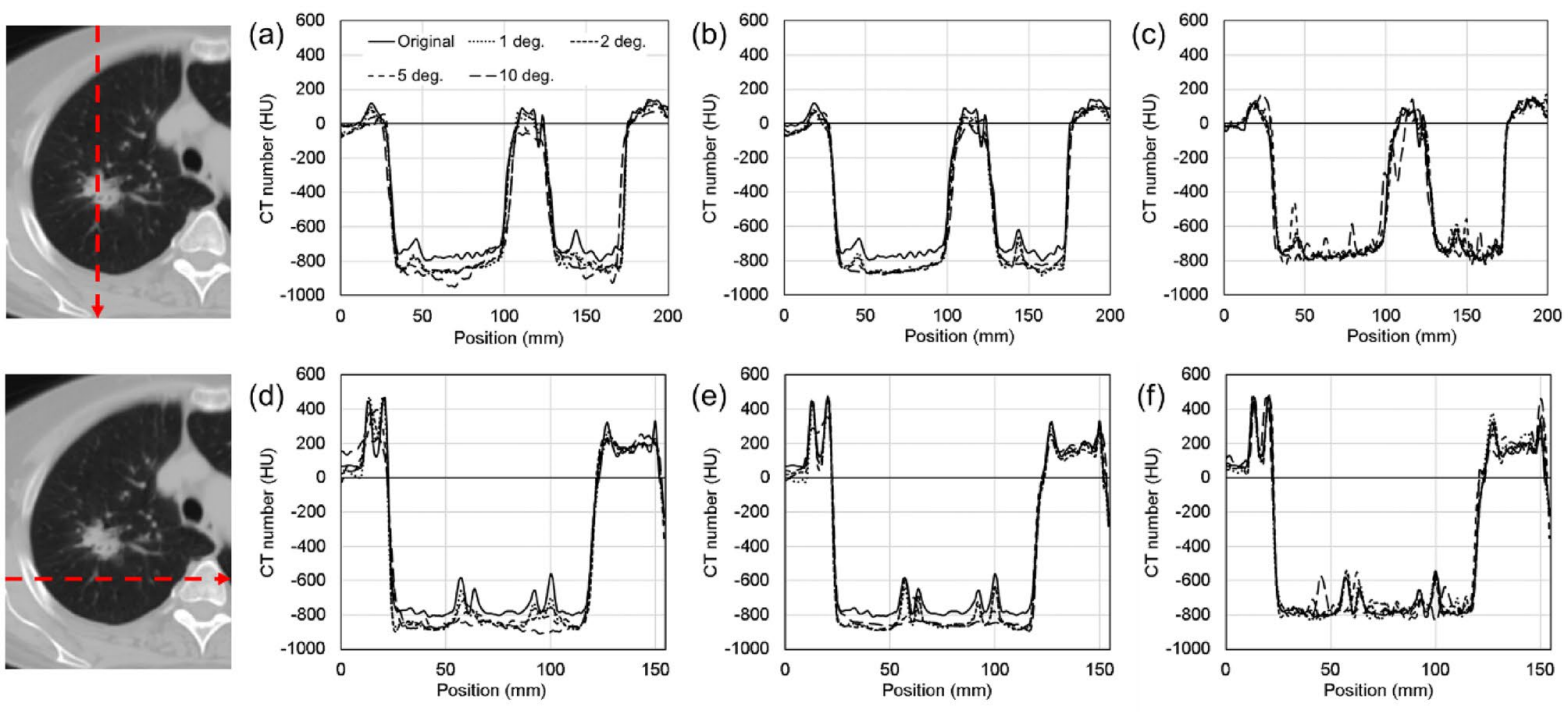
Line profiles of the CT value in each correction image using the deep learning models. (**a**–**c**) vertical profile direction and (**d**–**f**) horizontal profile direction. (**a**,**d**) Images corrected by the AE model, (**b**,**e**) images generated by the U-net model, and (**c**,**f**) images generated by the CGAN model.

Figure 6 shows the results of the artifact correction image in the sparse-view CT using each deep learning method. Figure 6a shows representative axial slice images with a dispersed projection angle of 1°. The lower images show subtracted images (the artifact correction image minus the original image), as shown by the absolute difference in CT values. Moreover, Figs. (b), (c), and (d) show the results for each corrected image with dispersed projection angles of 2°, 5°, and 10°, respectively. The simulated sparse-view CT image degraded the image quality, and large deviations in the CT values were observed in the subtraction image, particularly for dispersed projection angles greater than 5°. By contrast, the simulated sparse projection artifacts were small at dispersed projection angles of 1° and 2°. Through artifact reduction using each deep learning model, differences in CT values in the lung and air regions were observed in the subtraction image, as shown in the AE and U-Net results. Figure 7 shows the enlarged images of each artifact-reduced image. Moreover, Fig. 8 displays the line profiles of the CT value in each deep learning model. Image artifacts caused by sparse projections were reduced even when the dispersed projection angle was greater than 5°. However, the image resolution was significantly decreased, and image blurring occurred as shown in the results of the AE and U-Net models, resulting in missing fine structures within the lung region. Moreover, the low CT value, which was approximately − 800 hounsfield unit (HU) in the lung region, differed from the original images. By contrast, we observed that the CGAN model reduced the artifacts while maintaining the image resolution. However, the detailed shape of the tumor and spine changed, and it was not possible to completely restore the image at dispersed projection angles of 5° and 10°. Moreover, CT value profiles in the CGAN model had some hallucinated regions in the lung and tumor areas, particularly in the results for dispersed projection angles of 5° and 10°.

Tables 1 and 2 present the ME and MAE results compared with the original CT image. For large decimation projection angles, the CT values differed from those of the original images. The results of AE and U-Net show large deviations in each region; in particular, the lung and air regions had a difference of over 45 HU. For CGAN, the HU values were similar to those of the original image for each sparse projection angle; however, a slight deviation was observed in the bone region, and the differences were generally less than 10 HU. Although the HU values in each ROI approached the original image, hallucinated structures were generated.

**Table 1:** Mean error in the CT values of each site: lung, soft tissue, bone, and air regions. These values were calculated with respect to the CT numbers in the original images and are shown in terms of mean ± standard deviation for evaluation images.

	Interval of projection data acquisition angle			
	1°	2°	5°	10°
Lung				
With artifacts	− 0.08 ± 0.06	− 0.07 ± 0.13	− 0.07 ± 0.67	3.80 ± 3.51
AE	− 52.90 ± 0.84	− 48.70 ± 0.71	− 47.49 ± 1.73	− 75.42 ± 6.98
U-Net	− 54.01 ± 0.31	− 54.96 ± 0.45	− 54.89 ± 0.78	− 59.74 ± 2.24
CGAN	1.47 ± 0.20	− 0.85 ± 0.25	0.97 ± 1.70	− 5.38 ± 2.84
Soft tissue				
With artifacts	− 0.08 ± 0.09	− 0.09 ± 0.12	− 0.59 ± 0.55	− 2.38 ± 1.59
AE	− 17.28 ± 0.34	− 9.89 ± 0.61	− 5.81 ± 1.44	− 3.63 ± 2.12
U-Net	− 19.68 ± 0.28	− 25.55 ± 0.20	− 14.60 ± 1.08	− 21.09 ± 1.78
CGAN	2.98 ± 0.11	0.82 ± 0.14	0.32 ± 1.74	− 0.16 ± 1.93
Bone				
With artifacts	0.14 ± 0.20	− 0.33 ± 0.50	− 0.40 ± 3.07	− 6.38 ± 12.80
AE	− 10.57 ± 2.65	− 3.20 ± 3.10	− 8.75 ± 4.25	7.13 ± 10.25
U-Net	− 14.35 ± 1.27	− 20.62 ± 1.39	− 8.83 ± 2.55	− 0.85 ± 10.77
CGAN	3.26 ± 0.45	0.14 ± 0.74	4.15 ± 4.25	− 2.02 ± 11.55
Air				
With artifacts	− 1.30 ± 0.08	− 0.66 ± 0.24	12.20 ± 2.49	35.95 ± 1.55
AE	− 62.27 ± 0.24	− 62.47 ± 0.14	− 61.19 ± 0.46	− 58.75 ± 0.96
U-Net	− 61.95 ± 0.19	− 60.97 ± 0.21	− 60.48 ± 0.46	− 61.95 ± 0.48
CGAN	− 0.45 ± 0.09	− 1.13 ± 0.17	0.86 ± 0.50	1.23 ± 0.45

**Table 2:** Mean absolute error in the CT numbers of each site: lung, soft tissue, bone, and air regions. These values were calculated with respect to the CT numbers in the original images and are shown in terms of mean ± standard deviation for evaluation images.

	Interval of projection data acquisition angle			
	1°	2°	5°	10°
Lung				
With artifacts	0.08 ± 0.05	0.12 ± 0.09	0.49 ± 0.44	3.88 ± 3.44
AE	53.11 ± 0.84	48.89 ± 0.72	47.67 ± 1.74	75.72 ± 7.01
U-Net	54.22 ± 0.31	55.17 ± 0.45	55.11 ± 0.79	59.97 ± 2.24
CGAN	1.48 ± 0.20	0.85 ± 0.26	1.61 ± 1.05	5.40 ± 2.86
Soft tissue				
With artifacts	0.10 ± 0.07	0.12 ± 0.08	0.66 ± 0.46	2.51 ± 1.38
AE	17.35 ± 0.34	9.93 ± 0.62	5.83 ± 1.44	3.64 ± 2.13
U-Net	19.75 ± 0.28	25.65 ± 0.20	14.66 ± 1.08	21.18 ± 1.79
CGAN	2.99 ± 0.11	0.83 ± 0.14	1.38 ± 1.03	1.28 ± 1.39
Bone				
With artifacts	0.21 ± 0.10	0.48 ± 0.34	2.64 ± 1.39	11.89 ± 7.33
AE	10.61 ± 2.66	3.96 ± 1.94	8.78 ± 4.27	9.56 ± 7.83
U-Net	14.41 ± 1.27	20.71 ± 1.40	8.86 ± 2.56	9.35 ± 4.52
CGAN	3.27 ± 0.46	0.59 ± 0.43	4.17 ± 4.27	9.42 ± 6.36
Air				
With artifacts	1.30 ± 0.08	0.66 ± 0.25	12.25 ± 2.50	36.09 ± 1.56
AE	62.51 ± 0.24	62.71 ± 0.14	61.43 ± 0.46	58.98 ± 0.97
U-Net	62.20 ± 0.19	61.21 ± 0.21	60.71 ± 0.46	62.19 ± 0.48
CGAN	0.45 ± 0.09	1.13 ± 0.18	0.86 ± 0.50	1.23 ± 0.45

Table 3 presents the results in terms of the SSIM and PSNR for each dispersed projection angle with artifact reduction in each model. Through artifact reduction using the CGAN model, the SSIM and PSNR significantly improved for all sparse projection angles. By contrast, the SSIM values of AE and U-Net were less than 0.5, and the PSNR degraded compared to the original image at sparse projection angles of 1° and 2°.

**Table 3:** SSIM and PSNR values in each dispersed projection angle with artifact reduction by deep learning models. These values were calculated based on the original CT image. *p-values of < 0.005 were deemed significant for differences from the results of corresponding results of artifacts image.

	Interval of projection data acquisition angle			
	1°	2°	5°	10°
SSIM				
With artifacts	0.87 ± 0.02	0.58 ± 0.02	0.29 ± 0.02	0.17 ± 0.02
AE	0.40 ± 0.03*	0.38 ± 0.02*	0.37 ± 0.02*	0.31 ± 0.03*
U-Net	0.41 ± 0.03*	0.41 ± 0.03*	0.40 ± 0.03*	0.31 ± 0.02*
CGAN	0.94 ± 0.02*	0.90 ± 0.03*	0.87 ± 0.02*	0.79 ± 0.02*
PSNR				
With artifacts	33.6 ± 0.3	26.8 ± 0.3	19.7 ± 0.4	16.0 ± 0.5
AE	24.8 ± 0.1*	24.5 ± 0.1*	23.3 ± 0.2*	20.4 ± 0.4*
U-Net	24.9 ± 0.1*	24.8 ± 0.1*	24.0 ± 0.1*	21.1 ± 0.3*
CGAN	36.7 ± 0.7*	33.3 ± 0.4*	29.3 ± 0.4*	23.7 ± 0.6*

*p < 0.005, showing a significant difference from the artifact conditions.

## Discussion

Our study attempted to reduce image artifacts in sparse-view CT images using deep learning. Sparse-view CT reduces patient exposure dose, and artifact reduction is essential when applying this technique to clinical CT images. For the CGAN model, each loss function in Eq. (1) was updated by the object in Eq. (2), including generator and discriminator loss, thereby increasing the similarity with conditional label data. Therefore, the performance of the CGAN model, which was hypothesized to improve the accuracy of CT value reproducibility and the image quality index in terms of SSIM and PSNR, was evaluated for artifact correction using simulated sparse-view CT images.

As shown in Figs. 6 and 7, image quality degradation with sparse projection occurred in the simulated sparse-view CT, particularly at decimation angles of 5° and 10°, as shown in the subtraction image. As shown in the results of the AE model, the restoration of the decoding process was insufficient because of the suppression of the artifact region with a relatively high-contrast resolution component. This issue was observed at decimation angles of 5° and 10°. The U-Net model reduced artifacts while maintaining the image resolution at a decimation angle of up to 5°. However, partial over-smoothing was observed at the boundary between the adipose and muscle regions. This result shows the same tendency as that reported in previous studies^30,31^. Moreover, the lung and air regions were different from the original image in the artifact-reduced images generated by AE and U-Net, as shown in Fig. 8. The low-density CT value in the lung region tends to be smoothed with an excessively low CT value; therefore, the microvessel structure in the lung vanishes. This effect can be seen in the reconstructed image in Figs. 6 and 7, even for decimation angles of 1° and 2°. As shown in Tables 1 and 2, the ME and MAE exhibited large differences in the lung and air regions from the original image. The AE and U-Net set the loss function of MSE compared to the training data and learning with total variation regularization; therefore, these over-smoothing corrections and filling with a uniform value were possibly shown in the artifact-reduced images by AE and U-Net. This result could affect the accuracy of computational analyses using CT values in images, such as computer-assisted detection/diagnosis and radiation treatment planning. By contrast, the artifacts of sparse projection were corrected, and over-smoothing of the pixel value did not occur with the CGAN method. Therefore, ME and MAE were also low by almost under 5 HU in all regions, and the artifact-corrected image by CGAN was suitable for applying the computational analysis image. However, some hallucinated regions were observed in the lung region, as shown in the profile results in Fig. 8. CGAN generates similar images with learning features that are close to the condition images. The generator receives random noise as input, which adds an element of randomness to the generated data. Therefore, each iteration of the learning process provides different yet similar data, and caution must be exercised with the potential creation of a delicate structure that lacks actual existence. In lung CT images, these hallucinated regions may be misdiagnosed as microscopic tumors and pulmonary blood vessels. Therefore, it is difficult to use sparse projection images corrected by the CGAN model as diagnostic images. However, because the CT values within the region are close to the original image, it may be possible to use it for radiation treatment planning. Accurate CT values in each organ region are necessary to calculate dose distribution using CT images; however, this correction image with the CGAN model is not suitable for recontouring organ structures, including tumor regions.

Table 3 shows the results in terms of SSIM and PSNR for sparse projection and correction images, for each model. CGAN achieved the highest SSIM and PSNR values. An SSIM of over 0.8 and PSNR over 20 dB were accomplished even at the decimation angle of 10°. In a previous study on the image correction of sparse-view CT, the SSIM and PSNR were approximately 0.8 and 30 dB, respectively, using dual CNN-based methods^31^. Results achieved by CGAN were comparable with those of previous studies; therefore, the CGAN model can synthesize accurate images of the sparse-view CT. However, SSIM and PSNR significantly degraded for AE and U-Net because ME and MAE were large for these models.

The CGAN model significantly improved the image quality index in terms of SSIM and PSNR compared to the U-Net and AE models. With the addition of conditions and L1 norm regularization, CGAN significantly improved artifact correction for sparse-view CT and restored a synthetic image that is close to the original image. In this study, accurate restoration of image quality, including organ structure and CT values, was achieved using the CGAN model up to a decimation angle of 2°. Over a decimation angle of 5°, the details of the organ structures appeared to be transformed, and CGAN had limitations in terms of accurate restoration. However, the image similarity index, in terms of SSIM and PSNR, significantly improved using CGAN correction compared with the sparse projection image in all decimated angle cases. Therefore, the accurate restoration of pixel values in the lung region, soft tissue, and bones can enhance the accuracy of image registration using pixel value information and improve the accuracy of calculating the distribution of radiation doses in radiation therapy. Many groups have explored sinogram synthesis methods based on CNNs in the projection domain and proposed filling in missing view data in sinograms^32,33^. Our study applied reconstructed images as training and evaluation data; therefore, artifact correction was performed on the reconstructed CT images. Because there is no sinogram-based correction, our study has the advantage of not being affected by filter characteristics such as high-frequency enhancement by the FBP. As the image reconstruction process can be accelerated with direct correction in the reconstructed image, it is more practical in clinical practice. Moreover, implementing artifact correction directly on the reconstructed images is considered more practical and versatile because users cannot acquire sinograms directly from clinical CT scanners. However, focusing on the details of the tumor contour, the details of the tumor structure were distorted, limiting the complete reconstruction of the structure using the CGAN model. Moreover, unexpected hallucinate regions occurred when using the CGAN model, and it is imperative that we thoroughly evaluate the intended purpose of the generated images and strive for their practical use.

In previous studies, the compressed sensing (CS) method was applied to reconstruct CT images from sparse projection data^15,17^. This method formulates the reconstruction problem as a convex optimization problem with data fidelity and image sparsity, thereby promoting regularizer terms. A numerical solver iteratively solves the image reconstruction optimization problem to remove view angle undersampling-induced aliasing artifacts and correct the reconstructed image against the recorded data. However, the pixel value in each region tended to over smooth the value, which showed as patchy image. Zhang et al. developed the prior image-constrained compressed sensing (PICCS) method to prevent the occurrence of blurring, and severe patching appeared in the reconstructed image^16^. In this method, a prior image reconstructed using the FBP algorithm from the union of interleaved dynamic projection datasets was used to constrain the CS image reconstruction method. However, a prior image of the same slice position with full projection data is required, and our research cannot be applied because there is no process to acquire the same prior image. In recent years, combining the deep learning reconstruction method with the prior image-constrained CS (PICCS) algorithm has been proposed to improve the reconstruction accuracy for individual patients and enhance generalizability for sparse-view reconstruction problems^34^. In this method, the prior image was created using deep learning with the U-Net model, and it is possible that CGAN can be used instead of the existing model. Harms et al. proposed a paired cycle-GAN-based CBCT image correction method, which led to the accurate restoration of HU values and the removal of streaking and shading artifacts^25^. In this study, the residual network made it possible to create accurate synthetic CT (corrected CBCT) by learning specific differences between CBCT and CT. In our study, the CGAN model using paired supervised images can reduce non-locally streaking artifacts in sparse-view CT. Moreover, we compared the performance of artifact reduction between the conventional AE and U-net models and revealed the significance of image quality improvement for sparse-view CT. If we cannot acquire paired supervised original and sparse-view CTs, the cycle-GAN model is one of the adequate deep learning models to improve the image quality of the sparse projection. However, unsupervised learning may result in lower image synthesis accuracy than supervised learning^35,36^.

It is difficult to collect a large number of pixel-by-pixel paired CT images with sparse projections in a clinical CT unit because conventional CT equipment involves continuous rotational data acquisition. Therefore, in this study, many virtual sparse-view CT images were created from sufficiently projected CT images using computational simulations, and deep learning was performed using these images. CGAN needs to add a conditional label using paired images, and the effectiveness of image quality improvement is expected. In this study, the image evaluated by deep learning models was an artifact image generated by the computational simulation of sparse projection, and the correction effect for the artifacts caused by the actual sparse projection was not verified. However, because sparse projection is not possible with current clinical CT units, the CT data acquisition system needs to be modified to apply artifact correction methods with deep learning. We believe that our research findings can contribute to reducing radiation exposure and shortening imaging time (by reducing the projection data per phase owing to 4D reconstruction) in cone-beam CT images that can be acquired through sparse projection. Our study clarified the effect of image quality improvement for sparse-view CT using three deep learning models and revealed that the CGAN model can synthesize the most similar image, including consistency of CT values. For the clinical application of artifact correction of sparse-view CT images, it is necessary to evaluate the practicality of artifact correction using CGAN by verifying the accuracy of this learning model for actual sparse projection images in future studies. This study was limited to the use of diagnostic helical CT image, and clinical data will need to be generated from a real cone-beam CT system to clarify the contribution of this deep learning model.

## Conclusion

To suppress image artifacts in sparse-view CT, a deep learning model, CGAN, was constructed using artifact images created by computational simulation as training data, and its correction effect was compared and evaluated with that of other deep learning models. The CGAN model demonstrated high image reproducibility compared to AE and U-Net, as well as particularly accurate CT value restoration. However, over a decimation angle of 5°, the accuracy of reconstructing exact organ structures was limited, and unexpected structures could be generated.

## Data Availability

The datasets used and analyzed during the current study available from the corresponding author on reasonable request.

## Abbreviations

CT: Computed tomography
CBCT: Cone-beam computed tomography
CNN: Convolutional neural network
GAN: Generative adversarial network
CGAN: Conditional generative adversarial network
AE: Autoencoder
ROI: Regions of interest
ME: Mean error
MAE: Mean absolute error
SSIM: Structural similarity index
PSNR: Peak signal-to-noise ratio
HU: Hounsfield unit
